# Unpredictable Malnutrition and Short-Term Outcomes after Single Anastomosis Sleeve Ileal (SASI) Bypass in Obese Patients

**DOI:** 10.1155/2023/5582940

**Published:** 2023-06-19

**Authors:** Ayman Kamal, Mahmoud El Azawy, Tarik A. A. Hassan

**Affiliations:** Faculty of Medicine, Helwan University, Helwan, Egypt

## Abstract

**Objectives:**

The aim of this study is to present the clinical outcomes of SASI bypass as a treatment alternative for patients with morbid obesity.

**Methods:**

This study was a prospective follow-up of morbidly obese patients who underwent SASI bypass at Helwan University Hospital between March 1, 2019, and March 2020. The surgical procedure involved sleeve gastrectomy, followed by the anastomosis of the ileum, which was brought and hand-sewn 4 cm length side to side with the antrum, at a distance of 250 cm from the ileocecal valve. The data collected for the study included the resolution of comorbidities, incidence of gallstones, and one-year morbidity.

**Results:**

The mean age of the studied patients (*n* = 30) was 44.13 ± 8.9 years. The mean BMI of the studied patients was 47.3 ± 7.6 kg/ht^2^. All patients were morbidly obese for an average of 24 years. Postoperatively, 48% of the patients (*n* = 13) developed gallstones (GS), and the formation of GS was significantly higher in patients with longer durations of obesity (*P* = 0.009) and rapid weight loss. There was a significant decrease in the incidence of GS after 12 months postoperatively (*P* < 0.05). 63% of the patients (*n* = 19) had malnutrition, and 15 cases required revision due to the fear of further weight loss. Revision and malnutrition were significantly higher among male patients than female patients and among patients with longer durations of obesity (*P* ≤ 0.001).

**Conclusion:**

The SASI bypass may be an effective bariatric and metabolic surgery that can achieve satisfactory weight loss and improvement in medical comorbidities. However, our study highlights the potential risks of severe malnutrition and unpredictable weight loss; patient selection and duration of obesity may play a role in mitigating these risks.

## 1. Introduction

Single anastomosis sleeve ileal (SASI) bypass is a novel surgical technique that has emerged recently [1]. Santoro et al. introduced a new concept called the bipartition principle, which involves the early diversion of a portion of the ingested meal into the ileum, while the remaining portion of the meal continues through the normal pathway into the duodenum [1]. The bipartition procedure was devised mainly as a metabolic procedure to treat diabetes mellitus (DM). The bipartition principle assumes that patients with morbid obesity may have an excessive absorption of ingested nutrients in the proximal bowel and diminished distal absorption of these nutrients [2]. The bipartition mechanism is thought to counterbalance the digestive tract signaling abnormalities and correct the enterohormonal disturbance resulting in the improvement of blood sugar control [2]. Following surgery, patients with type 2 diabetes demonstrated an improvement and even remission of their DM [2, 3]. Regular and constant postoperative observation has been highly recommended as some patients may experience serious vitamin and mineral deficiencies. [4].

The aim of this study was to evaluate the feasibility and efficacy of the SASI technique in our hospitals.

### 1.1. Patients and Methods

This was a prospective study conducted at Helwan University Hospital in Egypt, which included 30 patients with morbid obesity who underwent SASI bypass at a single hospital from March 1, 2019, to March 2020.

The eligibility for bariatric surgery (BS) was determined based on the following criteria: a body mass index (BMI) ≥ 35 kg/m^2^ with one or more obesity-related comorbidities or a BMI > 40 kg/m^2^ without any coexisting comorbid conditions and those patients for whom the BS procedure would not pose an excessive risk [5].

Preoperative factors such as sex, BMI, diabetes mellitus, previous surgery, history of cholecystitis, white blood cell count, GB wall thickness, and presence of pericholecystic fluid had all been demonstrated to be predictive factors for surgical difficulty.

After the surgery, patients were monitored for symptoms or complications related to gallstones (GS) and the diagnosis was made through clinical examination, blood tests (complete blood count and liver function tests), and abdominal ultrasound (US). In addition, data on comorbidity resolution, one-year morbidity, and mortality were collected.

All patients were asked to sign an informed consent form before participating in the study, and the study was approved by the Research Ethical Committee, Helwan Faculty of Medicine.

Patients who had Mirizzi syndrome (excluded intraoperative), a history of calculous obstructive jaundice, or a history of upper abdominal surgery were excluded from the study. Patients with previous bariatric surgery or less than one year of follow-up were also excluded.

After meeting the inclusion criteria, all patients underwent the following procedures and assessments.

### 1.2. Preoperative Assessment

Demographics, anthropometric features, years of obesity, and comorbidities of patients were documented. Before surgery, patients underwent preoperative laboratory investigations including complete blood cell count; kidney, liver, and bleeding profile; and thyroid and cortisol panels. A cardiopulmonary work-up was also performed including ECG/ECCHO assessment and chest radiograph for all patients. Those at a high risk for pulmonary complications received a sleep study and pulmonary function tests. In addition, routine abdominal ultrasound was performed to detect any gallbladder pathology and measure the size of the liver. Preoperative anticoagulation was not used except in high-risk patients who had previous DVT.

### 1.3. Surgical Technique

During the surgery, the patients were placed in a supine position with reverse Trendelenburg tilt and were intubated. Pneumoperitoneum was established using a 10-mm umbilical visiport. The surgeon's instruments were inserted through 12- and 15-mm trocars on the right and left-middle clavicular lines, respectively. An additional 5-mm trocar was placed on the left anterior axillary line for assistance.

An oral Ryle's tube was inserted at the beginning to deflate the stomach. Dissection was started on the greater curvature, that is, 5 cm from the pylorus up to the cardio-esophageal junction until full mobilization of the gastric fundus was achieved. Once the greater curvature was liberated, a 36-French orogastric tube was inserted into the stomach and duodenum. The stomach was resected using endo-GIA linear staplers that were applied parallelly to the lesser curvature, starting from 3 to 5 cm from the pylorus and extending up to the angle of Hiss. The hemostasis and staple line were checked using methylene blue.

**Figure 1:**
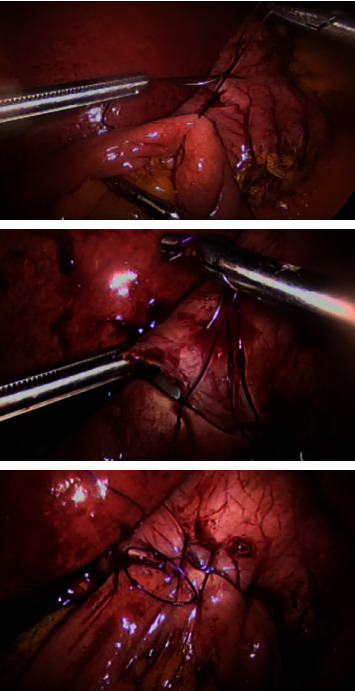
Hand-sewn anastomosis of the stomach and jejunum.

After the creation of the sleeved gastric tube, the patient's position was changed to the Trendelenburg position. The transverse mesocolon was pulled towards the head of the patient, and 250 cm of the ileum was measured from the ileocecal junction. A 4 cm antecolic side-to-side gastrojejunostomy was then performed at the anterior wall of the area between the antrum and the body of the stomach using hand-sewn PDS 2/0 sutures (see Figure 1). Full intestinal measurements were not performed. A leak test was performed by injecting 50–100 cc of methylene blue. The resected stomach was removed through the left midclavicular port, and the procedure was completed with a gastric tube having two outlets: one to the duodenum and the other to the ileum. Drains were left in place for 24 hours.

### 1.4. Postoperative Management

1. Patients were given a prophylactic dose of low molecular weight heparin 24 hours postoperatively for a duration of 14 days and were provided with elastic stockings during their hospital stay to prevent thromboembolism. On the second day after the surgery, patients were allowed to start consuming clear liquids. If the intraabdominal drain output was less than 50 ml of serosanguineous fluid, it was removed after 24 hours. However, in cases of unusual operative bleeding, a higher risk for postoperative bleeding, and complex operative cases, the drain was left in place and removed during the patient's first outpatient clinic visit.
2. All patients who did not experience any complications were discharged on the second day after their surgery. They were given instructions on their diet, physical activities, and medications, including multivitamins.
3. Patients were instructed to consume low-calorie clear liquids for one week followed by low-calorie semisolid foods for two to four weeks. They were then advised to gradually introduce a full diet in the form of a small frequent high protein diet. Multivitamin supplements were administered after 2 weeks for lifelong consumption.
4. During this waiting period, all patients underwent screening for biliary symptoms such as biliary colic, cholecystitis, acute cholangitis, obstructive jaundice, and biliary pancreatitis. This was performed through clinical examination and blood work (including total leukocytic count and liver function tests), and ursodeoxycholic acid was not used postoperatively.
5. Patients were followed up in the outpatient clinic (OPC) on a weekly basis during the first month after their surgery for an early detection of any postoperative complications, such as fever, collection, bleeding, or leakage. Subsequently, they were followed up at 2, 6, and 12 months after surgery to evaluate surgical outcomes such as BMI, fasting blood sugar, lipid profile, and indicators of nutritional complications such as plasma levels of albumin, hemoglobin, and calcium.
6. During the 12 months of follow-up, micromalnutrition was assessed by vitamin D levels less than 30 ng/mL, while macromalnutrition was assessed by hemoglobin levels less than 10 g/dL or albumin levels less than 3.5 g/dL.

## 2. Results

For the patients included in the study (*n* = 30), their ages ranged from 28 to 60 years with an average of 44.13 ± 8.9 years (mean ± standard deviation (SD)). The male-to-female ratio was 50% to 50%. The average BMI was 47.3 ± 7.6 kg/m^2^. All of the patients were morbidly obese for an average of 24 years (Tables 1 and 2).

The most significant comorbidity was hyperlipidemia in approximately two-thirds of the studied patients (70%), followed by diabetes mellitus (DM) and hypertension in 63.3% and 40% of the patients, respectively. Symptomatic gallstones (GS) were present in 10% of the studied patients (3 patients), and concomitant cholecystectomy (CC) was performed. Gastroesophageal reflux disease (GERD) was found in 26.7% of the cases (8 patients) (Table 3).

All of the studied patients stayed for one day postoperatively and required drains for 24 hours. The mean liver size of the studied patients (measured in MCL by ultrasound) was 17.5 ± 1.04. The duration of operations ranged from 47 to 120 minutes. Operation time was significantly longer when gallstone (GS) removal was performed (*p* value < 0.05). CC operations performed before bariatric surgery had no complications, while only 2 cases (6.7%) of operations resulted in complications of cerebral stroke, which improved with medical treatment (Tables 4 and 5).

Hypertension, GERD, and DM were completely cured with highly statistically significant differences, with *p* values of ≤0.001, ≤0.001, and ≤0.005, respectively (see Table 6).

Out of 27 patients, 13 (48%) had asymptomatic gallstones postoperatively. Gallstone formation was significantly higher in patients with a longer duration of obesity (*p* = 0.009) and was associated with rapid weight loss, as indicated by a lower BMI. No cases of gallstones were found in patients with a BMI of 37.8 ± 6.22 at 3 months postsurgery, whereas 4 cases (13.3%) were found in patients with a BMI of 33.3 ± 5.3 at 6 months, 7 cases (23.3%) were found in patients with a BMI of 28.7 ± 5.1 at 9 months, and 2 cases (6.7%) were found in patients with a BMI of 26.77 ± 4.5 at 12 months. There was a significant decrease in the incidence of gallstones after 12 months postoperatively (*p* value < 0.05) (Tables 7–10).

One patient developed new-onset GERD postoperatively, which was managed with cruroplasty and RY gastric bypass due to concurrent macromalnutrition. Nine patients (30%) experienced vomiting in the early weeks after surgery, but they all improved with medical management. Dumping syndrome was observed in 10 patients (33.3%), and all cases were resolved with dietary interventions led by nutritionists.

**Figure 2:**
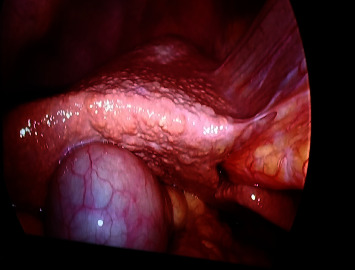
Laparoscopic observation of the liver in a patient with severe malnourishment.

Malnutrition was identified in 19 patients (63%), with 3 cases of micromalnutrition which improved with medical management and nutritionists, while 16 cases had macromalnutrition, in which one case improved with medical management and nutritionists and the other 15 patients (50%) required revision. One patient with short bowel syndrome and liver cirrhosis (Figure 2) was readmitted and treated with intravenous fluids and total parenteral nutrition before undergoing gastro-ileal bypass separation, and the others were managed initially by dietary advices and supplements, but their chemical profile (albumin and hemoglobin) did not improve, and they needed revision for fear of more loss of weight and severe protein intolerance, so we separated the anastomosis, and RY gastric bypass was performed in 12 and 2 cases, respectively, and all cases showed improvement. We preferred RY gastric bypass for patients with reflux symptoms with type c esophagitis. Six patients (20%) had steatorrhea, which was managed by nutritionists. The majority of patients (70%) experienced food intolerance, primarily severe protein intolerance, with 6 cases improving with dietary interventions, and the remaining cases requiring reoperation due to macromalnutrition. Male patients and those with longer duration of obesity had significantly higher rates of revision and malnutrition (*p* ≤ 0.001) (Tables 11–13).

### 2.1. Statistical Analysis

The data collected in this study were analyzed using Statistical Package of Social Services version 24 (SPSS). The results were presented in tables and graphs. Continuous quantitative variables, such as age, were expressed as the mean ± standard deviation (SD) and median (range), while categorical qualitative variables were expressed as absolute frequencies (number) and relative frequencies (percentage).

Appropriate statistical tests of significance were used after confirming normality. Results were considered statistically significant if the probability of the result occurring by chance was less than 0.05 (*p* < 0.05). A *p* value < 0.001 was considered highly statistically significant (HS), and a *p* value ≥ 0.05 was considered statistically insignificant (NS).

## 3. Discussion

The present study provides insight into the safety and effectiveness of primary SASI bypass in morbidly obese patients, particularly those with poor nutritional habits that may hinder weight loss or lead to weight regain after a restrictive bariatric procedure such as SG. The use of SASI bypass as an alternative to SG in these cases is an interesting approach and warrants further investigation. While the SASI bypass has been accepted as a standard procedure by the expert bariatric surgical consensus panel in 2018, but its lack of approval by IFSO highlights the need for continued evaluation and discussion of this procedure [6].

Kermansaravi et al. (2020) reported that the mean EWL% at six and twelve months after SASI bypass surgery was 67.8% and 86.2%, respectively [7]. Another study by Mahdy et al. (2016) showed EWL% of 75% and 90% at six and twelve months after SASI bypass, respectively [8]. Similarly, a separate study found EWL% of 46.2% and 72.6% at six and twelve months after SASI bypass, respectively [9]. A recent multicentric study also revealed an approximately 64% EWL one year after SASI [10], and Madyan et al. (2020) showed 44.3% and 65.2% EWL in twenty super obese patients after six and twelve months, respectively [11]. These findings indicate that SASI is a highly effective procedure for weight loss, particularly in the short term [12]. In our study, the mean BMI of the patients was 47.3 ± 7.6 kg/ht^2^, but it decreased to 33.3 ± 5.3 and 26.77 ± 4.5 at six and twelve months after SASI bypass, respectively.

In this present study, hypertension, GERD, and DM completely cured with highly statistically significant differences.

In the study by Kermansaravi et al. in 2020, it was reported that 100% of diabetic patients experienced complete remission or improvement after undergoing SASI bypass surgery [7]. Previous studies have also shown significant remission and improvement of type 2 diabetes mellitus (T2DM) (84–100%) within 1 year after SASI [8–10]. These studies suggest that SASI is a powerful tool for T2DM remission and improvement in short-term follow-ups and is considered to be more effective than Roux-en-Y gastric bypass and sleeve gastrectomy [12–14]. The reason for this superiority may be the rapid stimulation of GLP-1 and PYY secretion [8, 10, 15, 16].

According to Kermansaravi et al.'s study in 2020, remission of hypertension was found to be 86%, which is similar to the findings of the study by Mahdy et al. in 2016 [8], and they are better than other similar studies that reported remission rates of 36–57% [7, 8]. A noteworthy observation in the present study was the significant improvement in GERD symptoms following SASI bypass, with a rate exceeding 90%. This finding suggests that SASI bypass may correct the reflexogenic nature of SG [17]. The beneficial effect of SASI bypass on GERD symptoms may be attributed to the addition of gastro-ileal anastomosis, which can reduce intragastric pressure and can contribute to the amelioration of GERD symptoms [18].

The SASI procedure has raised concerns among some investigators regarding the potential for a high incidence of bile reflux due to the single-loop anastomosis between the stomach and the ileum [18]. However, a review by Emile et al. in 2021 found that the rate of bile reflux was only 3.4% [2]. In the present study, one patient (3.3%) developed de novo GERD, which was accompanied by macromalnutrition and necessitated cruroplasty with RYGB.

Obesity was found to be a major risk factor for the emergence of GS, which was reported in 22.8–43.6% of the morbidly obese undergoing BS [19].

Incidence of GS after BS varies but is close to 30% [5]. It has been reported that the risk of developing GS is high within 2 years after weight loss surgery. Alteration of the enterohepatic circulation and normal gallbladder physiology after gastric bypass can also lead to the development of cholelithiasis in 35% of the patients, while the incidence of symptomatic cholelithiasis in need of cholecystectomy after RYGB may diverge from 3 to 28% of patients [20].

According to a study by Elgohary et al. in 2021, the incidence of gallstones (GS) was higher in patients who underwent laparoscopic Roux-en-Y gastric bypass (LRYGB) (50%) and minigastric bypass (MGB) (40%) compared to laparoscopic sleeve gastrectomy (LSG) (18.8%) and single anastomosis duodeno-ileal bypass with sleeve gastrectomy (SASI) (33%) [21]. This suggests that certain types of weight loss surgery may be associated with a higher risk of developing GS than others. Also, SASI has shown to be effective in improving hypertension when compared to RYGB and SG [21, 22].

Another study by Worni et al. found that the amount of weight loss achieved after bariatric surgery was the most significant independent predictor of symptomatic GS [22]. Specifically, a weight loss of 25% at 10 months and 50% at 3 months was found to be a predictor of symptomatic GS after bariatric surgery.

In this study, it was found that 48% (13 out of 27) of patients developed asymptomatic gallstones after their surgery. The formation of gallstones was significantly higher in patients who had a longer duration of obesity, with a *p* value of 0.009. Patients who experienced rapid weight loss, indicated by a lower BMI, also had a higher incidence of gallstones. Specifically, at 6 months with a mean BMI of 33.3 ± 5.3, 4 cases (13.3%) developed gallstones. At 9 months with a mean BMI of 28.7 ± 5.1, 7 cases (23.3%) developed gallstones. Finally, at 12 months with a mean BMI of 26.77 ± 4.5, 2 cases (6.7%) developed gallstones. There was a significant decrease in the incidence of gallstones after 12 months postsurgery with a *p* value of <0.05 (Tables 7–10).

In our study, we found that 63% of the 19 patients had malnutrition, with 3 cases of micromalnutrition that improved with medical treatment and nutritionists and 16 cases of macromalnutrition. One case of macromalnutrition improved with medical treatment and nutritionists, and another case had short bowel syndrome and liver cirrhosis and was admitted for intravenous fluid and total parenteral nutrition. The latter patient was reoperated through separation of sleeve ileal anastomosis. We had a high threshold for revision in the remaining 14 patients for fear of further weight loss and severe protein intolerance, so 15 patients (50%) required revision. Of these, 12 cases had the anastomosis separated, and 2 cases had a RY gastric bypass. All cases improved following the revisions.

Moreover, we observed that revision and malnutrition were significantly higher among males than females and were more common in patients who had been obese for longer periods of time (*p* ≤ 0.001) (please refer to Tables 11–13 for further details).

In contrast, Tarnowski et al.'s study in 2022 reported only one case of hypoalbuminemia caused by short bowel syndrome. The patient underwent reoperation, during which the sleeve ileal anastomosis was separated. The authors concluded that the complication was due to improper anastomosis location and obstructed passage through the common limb. This finding suggests that the SASI bypass is relatively easy to deanastomose and can be converted into a restriction-only sleeve gastrectomy [23]. In their study, Tarnowski et al. used a 300 cm ileal loop from the ileocecal region as a common limb, which is longer than the technique used in our study. They also employed a linear stapler to create a sleeve ileal side-to-side anastomosis, located 6 cm proximally from the pylorus, and enrolled patients with a body mass index (BMI) between 35 and 39.9 kg/m^2^, which is lower than that in our study. Moreover, all of their patients were females.

The SASI bypass, which is based on the physiological principles of sleeve gastrectomy and ileal transposition, was first described by Mahdy et al. in 2016 [8]. Our study utilized a technique that is essentially the same as that reported by Mahdy, with a few technical differences. Specifically, we created a shorter common limb by performing the anastomosis 250 cm from the ileocecal region, and we hand-sewed a 4 cm ileogastric anastomosis. In addition, we began the greater curvature division 5 cm from the pylorus using a 36-Fr bougie. It is worth noting that 50% of our patients were male, and the mean BMI of the study population was 47.3 ± 7.6 kg/m^2^. All of the patients in our study had been morbidly obese for an average of 24 years.

Kristensson et al. study in 2020 found that individuals with early-onset obesity experienced slightly greater reductions in body weight and energy intake after bariatric surgery compared to those with late-onset obesity. This may be due to longer exposure to obesity, greater genetic influence, or an earlier establishment of an unhealthy lifestyle. The study also revealed small but statistically significant differences in weight loss among different subgroups after bariatric surgery, with the largest reductions observed in those with obesity aged 20 years. Overall, the study suggests that the age of onset of obesity may be an important factor to consider when assessing weight loss outcomes after bariatric surgery [24].

According to a study conducted by Emile et al. in 2021, the length of the common limb varied in different studies. Seven studies reported a length of 250 cm, while three studies reported a length of 300–350 cm [2, 9, 11, 25]. The study found that using a longer common limb was associated with a lower percentage of excess weight loss (%EWL) after 6 months (56 ± 11.7 vs. 60 ± 11.8, *p* < 0.0001) and fewer complications (3.9% vs. 14.2%, *p* = 0.0003). Moreover, the study found that using a 4-cm anastomosis was associated with a significantly higher %EWL. The study also recorded complications in 116 patients (12.3%), with hypoalbuminemia occurring in 12 patients.

In another study by Mohamed Khalaf, only one patient (0.3%) had severe protein-energy malnutrition and required reversal. This patient had a shorter common limb of 250 cm. Overall, the study highlights the importance of considering the length of the common limb in bariatric surgery to minimize complications and improve patient outcomes [26].

The variation in the %EWL after SASI bypass may be due to differences in patients' baseline characteristics or technical variations among the studies. It was notable that the outcome of SASI bypass was affected by technical aspects of the procedure, namely, the common limb length and anastomosis size. Patients with a common limb measuring 300–350 cm had less weight loss at 6 months and less complications, which is reasonable because a longer common limb is associated with less malabsorption. On the other hand, a larger anastomosis size was associated with a greater weight loss.

The standardization of the SASI bypass procedure was recently recommended by a consensus meeting, which proposed the following criteria: the width of the residual stomach should be 3 cm, the size of the gastric pouch should range from 150 to 250 cc, the gastro-ileal anastomosis should be situated 2–6 cm away from the pylorus, with a diameter of no more than 3 cm, and the common limb length should be 300 cm. This consensus was reached based on expert opinion and is intended to ensure consistency and optimal outcomes of the SASI procedure [27]. The main drawback of our analysis is the small study group, not allowing us to reach independently to any conclusions to propose recommendations. We would like to state, however, besides the technical precaution to avoid adverse effects such as excessive weight loss and protein malnutrition, we observed in our study that revision and malnutrition were significantly higher among males than females which may be justified by the total energy intake which is more in males [24] and higher in patients with longer years of obesity *P* = ≤0.001 (Tables 11–13).

## 4. Conclusion

In conclusion, SASI bypass appears to be an effective procedure for the treatment of morbid obesity and its related comorbidities. However, it is important to note that protein malnutrition and excessive weight loss are potential complications that may require further surgical intervention. To minimize these risks, continuous refinement of the technique is necessary and special consideration should be given to patients with longer exposure to obesity, especially males. In addition, it is worth noting that SASI bypass can be easily reversed to a sleeve gastrectomy if needed. Nonetheless, further studies are needed to fully evaluate the long-term outcomes and potential risks associated with this procedure.

## Figures and Tables

**Table 1:** Demographic details of the sample population studied.

Demographic data	Patients (*N* = 30)	
	No.	%
*Sex*		
Male	15	50.0
Female	15	50.0
*Age (years)*		
Mean ± SD	44.13 ± 8.9	
Median (range)	41 (28–60)	

**Table 2:** Anthropometric characteristics of the sample population studied.

Item	Patients (*N* = 30)
*Anthropometric data*	
* Weight (kg)*	
Mean ± SD	143.6 ± 31.5
Median (range)	130 (95–190)
* Height (cm)*	
Mean ± SD	165 ± 17.09
Median (range)	165 (127–197)
* BMI (kg/ht* ^*2*^)	
Mean ± SD	47.3 ± 7.6
Median (range)	47 (39–65)
* Obesity years*	
Mean ± SD	24.10 ± 9.8
Median (range)	26 (7–40)
*Previous pregnancy (N* *=* *15)*	
Mean ± SD	3.33 ± 1.8
Median (range)	4 (0–6)

**Table 3:** Patient clinical data and pre-existing conditions in the study.

*‡* Clinical data	Patients (*N* = 30)	
	No.	%
*Comorbidities*		
Smokers	8	26.7
Gallstones	3	10.0
Hypertension	12	40.0
DM	19	63.3
Hyperlipidemia	21	70.0
GERD	8	26.7

*‡*Multiple comorbidities.

**Table 4:** Operative duration among the sample population studied.

Operation time	Without GS	With GS	Test	*P* value
*Operation time*				
Mean ± SD	66.59 ± 14.7	120 ± 0	−18.80	≤0.001^*∗*^ (HS)
(Range)	(74–90)	120 (120–120)		

^*∗*^ *p* value <0.05 is significant. HS: highly significant.

**Table 5:** Complications after surgery in patients included in the study.

*‡* Complications	Patients (*N* = 30)	
	No.	%
*Complication of CC (N* *=* *3)*		
No	3	100.0
Yes	0	0.0
*Complication of operation (N* *=* *30)*		
No	28	93.3
Yes	2	6.7

**Table 6:** Comorbidities following surgery in patients included in the study.

Variables	Before		After		*P* value
	No	%	No	%	
*Hypertension*					≤0.001^*∗*^ (HS)
Not present	0	0.0	12	100.0	
Present	12	100.0	0	0.0	
*DM*					≤0.001^*∗*^ (HS)
Not present	0	0.0	19	100.0	
Present	19	100.0	0	0.0	
*GERD*					0.039^*∗*^ (S)
Not present	22	73.3	29	96.7	
Present	8	26.7	1	3.3	

McNemar test. ^*∗*^Statistical significance.

**Table 7:** Incidence of postoperative gallstones among the sample population studied.

Gall stones	US-3 months	US-6 months	US-9 months	US-12 months	Test	*P* value
Free	30 (100%)	26 (86.7%)	23 (76.7%)	28 (93.3%)	8.23	0.041^*∗*^ (S)
Yes	0 (0%)	4 (13.3%)	7 (23.3%)	2 (6.7%)		

^*∗*^ *p* value <0.05 is significant. S: significant.

**Table 8:** BMI variation after surgery in patients included in the study.

BMI	BMI-3 months	BMI-6 months	BMI-9 months	BMI-12 months	Test	*P* value
Mean ± SD	37.8 ± 6.22	33.3 ± 5.3	28.7 ± 5.1	26.77 ± 4.5	84.6	≤0.001^*∗*^ (HS)
(Range)	31–55	27–47	22–38	19–37		

^*∗*^ *p* value <0.05 is significant. S: significant.

**Table 9:** Time to postoperative gallstone occurrence among the sample population studied.

Items	Studied patients (*N* = 13)
*Time to gallstones*	
Mean ± SD	8.54 ± 2.06
Median (range)	9 (6–12)
*BMI at GS*	
Mean ± SD	27.15 ± 2.03
Median (range)	27 (23–29)

**Table 10:** Relationship between gallstone occurrence and certain other variables among the sample population studied.

Variables	GS free	GS formation	*P* value
Age	43.82 ± 10.26	44.54 ± 7.18	0.801
Previous pregnancy	3.09 ± 2.07	4 ± 1.15	0.426
Obesity years	20.06 ± 10.64	29.3 ± 5.44	0.009^*∗*^
BMI before operation	48.59 ± 6.59	45.7 ± 8.9	0.348
BMI at 3 months	38.824 ± 3.77	36.6 ± 8.46	0.014^*∗*^
BMI at 6 months	34.29 ± 3.8	32.08 ± 6.8	0.008^*∗*^
BMI at 9 months	30.47 ± 4.6	26.3 ± 4.9	0.024^*∗*^
BMI at 12 months	28.65 ± 4.15	24.3 ± 4.07	0.049^*∗*^

Data expressed in mean ± SD.

**Table 11:** Complications after surgery in patients included in the study.

Complications	Patients (*N* = 30)	
	No.	%
GERD	1	3.3
Revision	15	50.0
Vomiting	9	30.0
GS (*N* = 27)	13	48.1
Dumping	10	33.3
Malnutrition	19	63.3
Steatorrhea	6	20.0
Protein intolerance	21	70.0

**Table 12:** Gender-based differences in malnutrition among the sample population studied.

Variables	Male		Female		*P* value
	No	%	No	%	
*Revision*					≤0.001^*∗*^ (HS)
Not present	2	13.3	13	86.7	
Present	13	86.7	2	13.3	
*Malnutrition*					0.021^*∗*^ (S)
Not present	2	13.3	9	60.0	
Present	13	86.7	6	40.0	

Chi-square test. ^*∗*^Statistical significance.

**Table 13:** Association between malnutrition and duration of obesity in study participants.

Obesity years	Not present	Present	*P* value
	*Revision*		
Mean ± SD	18.6 ± 10.6	29.6 ± 4.7	0.001^*∗*^ (S)
Median (range)	14 (7–40)	30 (20–35)	

	*Malnutrition*		
Mean ± SD	15.5 ± 7.2	29.05 ± 7.5	≤0.001^*∗*^ (HS)
Median (range)	12 (7–25)	30 (14–40)	

Mann–Whitney test. ^*∗*^Statistical significance.

## Data Availability

The data used to support the findings of this study are included within the article.
